# Hospital Productivity After Data Breaches: Difference-in-Differences Analysis

**DOI:** 10.2196/26157

**Published:** 2021-07-06

**Authors:** Jinhyung Lee, Sung J Choi

**Affiliations:** 1 Department of Economics Sungkyunkwan University Seoul Republic of Korea; 2 Samsung Advanced Institute for Health Sciences & Technology Sungkyunkwan University Seoul United States; 3 School of Global Health Management and Informatics College of Community Innovation and Education University of Central Florida Orlando, FL United States

**Keywords:** cybersecurity, data breach, health information technology, health information, hospital data breach, hospital productivity, information technology, privacy

## Abstract

**Background:**

Data breaches are an inevitable risk to hospitals operating with information technology. The financial costs associated with data breaches are also growing. The costs associated with a data breach may divert resources away from patient care, thus negatively affecting hospital productivity.

**Objective:**

After a data breach, the resulting regulatory enforcement and remediation are a shock to a hospital’s patient care delivery. Exploiting this shock, this study aimed to investigate the association between hospital data breaches and productivity by using a generalized difference-in-differences model with multiple prebreach and postbreach periods.

**Methods:**

The study analyzed the hospital financial data of the California Office of Statewide Health Planning and Development from 2012 to 2016. The study sample was an unbalanced panel of hospitals with 2610 unique hospital-year observations, including general acute care hospitals. California hospital data were merged with breach data published by the US Department of Health and Human Services. The dependent variable was hospital productivity measured as value added. The difference-in-differences model was estimated using fixed effects regression.

**Results:**

Hospital productivity did not significantly differ from the baseline for 3 years after a breach. Data breaches were not significantly associated with a reduction in hospital productivity. Before a breach, the productivity of hospitals that experienced a data breach maintained a parallel trend with control hospitals.

**Conclusions:**

Hospital productivity was resilient against the shocks from a data breach. Nonetheless, data breaches continue to threaten hospitals; therefore, health care workers should be trained in cybersecurity to mitigate disruptions.

## Introduction

Data breaches are an inevitable risk to hospitals operating with information technology (IT). The US Department of Health and Human Services (HHS) defines a data breach as the impermissible use or disclosure of protected health information [1] and can be categorized as follows: theft, loss, unauthorized access or disclosure, improper disposal, hacking or IT incident, and unknown or other breaches. In the Healthcare Information and Management Systems Society 2019 Cybersecurity Survey, more than 80% of responding hospitals have reported that they experienced a significant security incident in the past 12 months [2].

Another growing cybersecurity threat to hospitals is ransomware attacks. Ransomware denies users the access to data by encrypting the data with a key known only to the attacker [3]. The attacker demands a ransom payment in exchange for the key to decrypt the user’s data. In one recent case, a hospital was forced to pay US $17,000 to regain access to its system. California-based Hollywood Presbyterian Medical Center reportedly experienced a malware attack, and employees stated that they were unable to access certain parts of the hospital network [4]. In a more severe case, University of California San Francisco paid over US $1 million to hackers to regain access to its system [5].

The Health Information Technology for Economic and Clinical Health Act regulates the notification of health information breaches in the United States. This act requires health care providers and entities covered by the Health Insurance Portability and Accountability Act of 1996 to notify a breach of protected health information, which affects more than 500 individuals to those affected individuals, HHS, and sometimes the media [1]. HHS maintains a public database called Breach Portal: Notice to the Secretary of HHS Breach of Unsecured Protected Health Information, which publishes the reported health data breaches submitted from October 2009 to the present [6].

Recovering from data breaches and ransomware attacks is costly for hospitals. Data breach remediation efforts were associated with lower hospital quality, including increased time-to-electrocardiogram and an increased 30-day acute myocardial infarction mortality rate [7]. In 2019, the average total cost of a data breach for all industries globally was US $3.92 million, and it took organizations an average of 279 days to identify and contain a breach. The average total cost of a data breach for all industries in the United States was US $8.19 million, which was more than 2-fold the global average [8]. The total costs include notification costs, productivity losses, re-establishing the image of the company, infrastructure costs, and repetition of work. The cost of a data breach is different across industries. The actual cost per breached record averages out at US $242 per record in the United States, and US $150 globally [8]. In the US health care industry, per-record breaches cost an average of US $429 [8]. Global losses from security breaches are forecasted to double from US $3 trillion per year in 2015 to US $6 trillion per year in 2021 [9]. In addition, breached hospitals potentially face investigation, fines, and several years of monitoring by the Office for Civil Rights (OCR) [10].

The additional costs associated with data breaches and their remediation has adverse implications for hospital productivity. The productivity of a firm is typically measured as the value of goods and services produced per unit of labor and capital input. For hospitals, productivity is the value of health care goods and services, such as pharmaceuticals and surgeries, per health care input [11-14]. The financial costs and regulatory burden associated with a data breach may divert resources away from patient care, thus negatively affecting hospital productivity. Disruptions in health IT systems after a breach may disrupt or delay the workflow of clinicians [7], thus negatively affecting hospital productivity. Employee layoffs and turnovers resulting from a breach are another factor that may reduce productivity [15]. Breach remediation required by the OCR, including changes to the health IT system and staff training, may take years to complete. Such an oversight by the OCR, which changes hospital policies and processes may disrupt hospital productivity in the long term. Organizational culture set by hospital administrators may have a strong influence on the productivity and security practices of the staff. Thus, hospitals with poor organizational culture may be involved in a breach and have poor productivity.

Despite the increasing importance of cybersecurity, little is known about its effects on hospital-level productivity. Health IT systems are intended to improve hospital productivity by reducing human error, but data breaches may have the unintended consequence of disrupting hospital productivity. Thus, in this study, we aimed to investigate the relationship between data breaches and hospital productivity by using data from California hospitals from 2012 to 2016. We hypothesized that data breaches may increase hospital costs and disrupt provider workflow, thus decreasing hospital productivity. We compared the productivity of the hospitals that experienced a data breach against control hospitals and investigated whether hospital productivity was significantly different for the breached hospitals before and after a breach.

## Methods

### Empirical Model

After a data breach, the resulting regulatory enforcement and remediation is a shock to a hospital’s patient care delivery. Therefore, hospital data breaches can be modeled as a natural experiment to understand the relationship between data breaches and productivity. The association between hospital data breaches and productivity was estimated using a generalized difference-in-differences model with multiple prebreach and postbreach periods [16]. This model for an event study is a widely used approach to model observational data in the health economics literature.

We used the reported information on breaches as collected by HHS to create a panel of hospital-year observations from 2012 to 2016. Our model estimates the changes in productivity associated with hospitals that experienced a breach, controlling for hospital financial characteristics including total assets, total labor, IT capital, IT labor, bed size, and time trends. The model assumes that the breached hospitals would have followed a trend parallel to that of the control group if they had not been breached.

For a hospital in a given year, the dependent variable is the log of productivity measured as value added. Value added is defined as operating revenues’ lesser intermediate inputs. Intermediate inputs include surgical supplies, linens, clothing, and other material inputs [11]. Financial control variables included the log of total capital, total labor, IT capital, and IT labor. Total capital assets include current assets, property, plant and equipment, intangible assets, assets whose use is limited, and other assets. Total labor (non-IT) is defined as the total conventional salaries, wages, employee benefits, and professional fees excluding any costs related to IT labor. IT capital is a summation of four components: purchased services, leases and rentals, other direct expenditure, and physical capital. IT labor is the summation of salaries and wages, employee benefits, and professional fees associated with data processing. For hospital control variables, we included the number of licensed beds and case mix index of a given hospital. For breach control variables, we included breach type and breach location. In addition, ownership, teaching status, and rural status were included in the descriptive summary, but they were omitted from fixed effects regression because they were time-invariant variables. Finally, the model included year fixed effects and hospital fixed effects. Assuming that hospitals’ administration does not change in the short term, hospital fixed effects serve to control for the unobserved time-invariant hospital organizational culture that may be correlated with both breaches and productivity.

For the treatment, dummy hospitals were categorized into two groups: never breached (control) and breached. Moreover, the breached hospitals experienced their specific breach events at different timepoints. The difference-in-differences model was specified to capture changes in value added at –3, –2 –1, 0, +1, +2, and +3 years relative to the hospital-specific year of the data breach. The year of the breach was set as the reference category. For example, a hospital that was breached in 2014 was coded as –2 in 2012, –1 in 2013, +1 in 2013, and +2 in 2014. The coefficients on the event time dummies captured the changes associated with value added at a given timepoint.

The model assumed that a breach was a one-time event. Multiple breaches within a year are a possibility, but we did not find any hospitals that experienced multiple breaches in our sample. The difference-in-differences model was estimated using fixed effects regression. SEs were robust to heteroskedasticity and allowed for within-hospital correlation analysis. Statistical analysis was performed using Stata (version 15, StataCorp) [17].

### Data

Breach data and California Hospital financial data were utilized in this study. Breach data published by HHS were used to identify hospital data breaches by hospital name and the date of the breach report [6]. All types of breaches were included (ie, theft, unauthorized access or disclosure, hacking or IT incident, improper disposal, and loss). Only breaches affecting 500 or more individuals were observed in our data; therefore, HHS data do not provide an exhaustive list of all hospital data breaches.

The California Office of Statewide Health Planning and Development (OSHPD) publishes audited financial data from approximately 450 participating nonfederal hospitals licensed by the state. Financial disclosure reports are filed annually by each licensed hospital. OSHPD data provided hospital characteristics and financial variables [18]. Hospital data breaches in the HHS data were merged with OSHPD hospital financial data in accordance with the hospital name and year. OSHPD provides a directory of hospitals and their business names and aliases, which uniquely identify each hospital. However, the HHS data do not provide a standard hospital identifier; thus, some breaches may have been merged incorrectly.

The study sample included general acute care hospitals from 2012 to 2016. For data consistency, hospitals whose financial statements spanned less than 1 year were excluded from the study. Breach activity prior to our study period could influence the response period assessed herein. Thus, hospitals that experienced a breach in the 2 years before our study period (2010 and 2011), were excluded for data consistency. Furthermore, all financial variables were trimmed at the top 1% to exclude outliers. The resulting study sample was an unbalanced panel of hospitals with 2610 unique hospital-year observations. Data breaches were reported by 31 hospital-year observations. The breached group had 205 hospital-years, and the control group had 2405 hospital-years.

## Results

### Descriptive Statistics

Descriptive statistics are summarized by breach status in Table 1. Hospital year observations were categorized as breached and never breached (control) groups. The number of hospital years was 205 in the breached group and 2405 in the never breached group. The breached group was larger with, on average, more than 2-fold the value added compared to the control group (US $429.4 million vs US $189.55 million, respectively). The breached group had almost 3-fold the total assets (US $685.06 million vs US $254.45 million, respectively) and more than 2-fold the labor spending (US $387.17 million vs US $169.84 million, respectively) than the control group. The breached group spent almost 3-fold more on health IT capital (US $32.43 million vs US $10.83 million, respectively) and spent almost 4-fold more on health IT labor (US $8.54 million vs US $2.20 million, respectively). The breached hospitals were more likely to be larger in bed size (348.8 vs 225.4, respectively) and higher in the case mix index (1.32 vs 1.27, respectively), less likely to be not-for-profit hospitals (43.41% vs 63.49%, respectively), and more likely to be public hospitals (26.34% vs 13.68%, respectively) and teaching hospitals (60.98% vs 6.65%, respectively).

**Table 1:** Descriptive summary of breached and never breached (control) hospitals.

Variables		Breached (n=205)	Never breached (n=2405)
**Continuous variables: financial variables in US $ (million), mean (SD)**			
	Value-added operating revenue	429.40 (507.97)	189.55 (173.54)
	Total assets	685.06 (916.47)	254.45 (323.21)
	Total labor	387.17 (413.95)	169.84 (148.41)
	Information technology capital	32.43 (71.58)	10.83 (19.07)
	Information technology labor	8.54 (15.69)	2.20 (4.12)
	Licensed beds	348.80 (211.11)	225.40 (158.10)
	Case mix index	1.32 (0.38)	1.27 (0.36)
**Categorical variables: ownership, n (%)**			
	Investor-owned hospitals	62 (30.24)	549 (22.83)
	Not-for-profit hospitals	89 (43.41)	1527 (63.49)
	Public hospitals	54 (26.34)	329 (13.68)
	Teaching hospitals	125 (60.98)	160 (6.65)

A comparison of the financial characteristics of breached and control hospitals between 2012 and 2016 is shown in Table 2. The breached group had a higher growth rate of value added, total assets, and total labor than the control group between 2012 and 2016 (128.27% vs 115.81% for value added, 128.38% vs 121.35% for total assets, and 117.24% vs 111.43% for total labor, respectively). The breached group had a higher growth rate than the control group in IT capital (186.69% vs 178.96%, respectively) and in IT labor (183.96% vs 123.82%, respectively) from 2012 to 2016. The breached group had a higher growth rate in licensed beds (100.39% vs 98.73%, respectively) between 2012 and 2016.

Individuals affected by a breach, breach type, and breach location among breached hospitals are summarized as follows. The mean number of individuals affected by a breach was 136,613. The proportion of breach types indicated that data theft was the most common breach type (65.85%), followed by unauthorized access, loss, or other breach types (22.00%), further followed by hacking or IT incidents (11.71%). The proportion of breach location indicated that desktop computers or laptops were the most common breach locations (51.22%), followed by network servers, papers, films, or other sources (36.1%), further followed by electronic medical records (12.68%).

**Table 2:** Descriptive summary of breached and never breached (control) hospitals between 2012 and 2016.

Variables			Breached (n=205)				Never breached (n=2405)		
		Mean (SD)		2016 vs 2012, %		Mean (SD)			2016 vs 2012, %
**2012, US $ (million)**									
	Value added	422.06 (494.78)		128.27	193.19 (172.91)			115.81	
	Total assets	659.66 (870.55)		128.38	262.89 (316.28)			121.35	
	Total labor	388.65 (416.00)		117.24	174.22 (151.02)			111.43	
	Information technology capital	29 (71.28)		186.69	9.71 (13.33)			178.97	
	Information technology labor	7.23 (11.52)		183.96	2.19 (3.33)			123.83	
	Licensed beds	346.30 (211.13)		100.39	226.96 (160.26)			98.73	
**2016, US $ (million)**									
	Value added	541.38 (634.86)		N/A^a^	223.73 (206.48)			N/A	
	Total assets	846.88 (114.84)		N/A	319.02 (437.35)			N/A	
	Total labor	455.64 (485.39)		N/A	194.15 (166.60)			N/A	
	Information technology capital	54.14 (98.85)		N/A	17.37 (36.87)			N/A	
	Information technology labor	13.30 (23.69)		N/A	2.72 (6.30)			N/A	
	Licensed beds	347.65 (207.02)		N/A	224.08 (153.52)			N/A	

^a^N/A: not applicable.

### Regression Results

**Figure 1:**
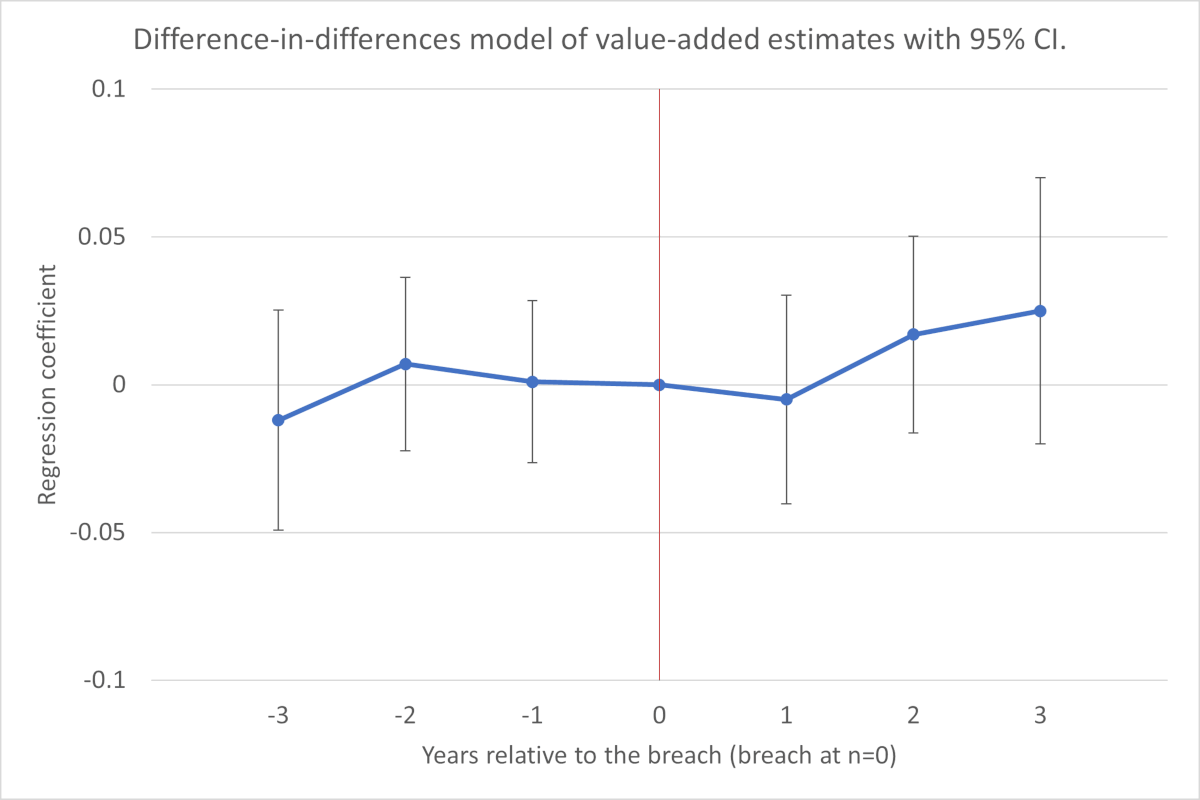
Difference-in-differences model of value-added estimates with 95% CI.

We estimated the change in value added associated with the years before and after a breach while controlling for hospital assets, labor, IT assets, IT labor, number of beds, case mix index, breach type, breach location, time trends, and hospital fixed effects. The regression coefficients are listed in Table 3 and visualized in Figure 1. We found that productivity remained practically unchanged before and after a breach relative to baseline, with constant observable time-varying covariates, time trends, and hospital fixed effects. Log-transformation of the dependent variable yielded regression coefficients that can be interpreted as multiplicative changes after exponentiation. Specifically, value added was associated with a 0.5% reduction [exp(-0.005)=0.995; *P*=.78] at 1 year after a breach, but the change was not significant. Furthermore, value added was associated with a 1.7% increase [exp(0.017)=1.017; *P*=.32] at 2 years after a breach, but the change was not significant. Moreover, value added was associated with a 2.5% increase [exp(0.025)=1.025; *P*=.28] at 3 years after a breach, but the change was not significant.

**Table 3:** Difference-in-differences model estimates for value added.

Breach parameters		Coefficient (SE)	*P* value
**Breach time for which ln (revenue) was calculated (reference=0)**			
	–3	–0.012 (0.019)	.53
	–2	0.007 (0.015)	.64
	–1	0.001 (0.014)	.94
	1	–0.005 (0.018)	.78
	2	0.017 (0.017)	.32
	3	0.025 (0.023)	.28
	Total assets	0.055 (0.016)	.001
	Total labor	0.600 (0.064)	<.001
	Information technology capital	0.045 (0.007)	<.001
	Information technology labor	0.007 (0.003)	.02
	Number of beds	0.091 (0.043)	.04
	Individuals affected	0.000 (0.000)	.27
	Case mix index	0.126 (0.079)	.11
**Breach type for which ln (revenue) was calculated**			
	Hacking or information technology incident (reference)	N/A^a^	N/A
	Data theft	0.148 (0.035)	<.001
	Unauthorized access, loss, or other	0.108 (0.021)	<.001
**Breach location for which ln (revenue) was calculated**			
	Desktop computer or laptop (reference)	N/A	N/A
	Electronic medical record	0.070 (0.033)	.04
	Network server, papers, films, or others	0.099 (0.020)	<.001
**Year for which the ln (revenue) was calculated (reference=2008)**			
	2009	0.024 (0.012)	.04
	2010	0.042 (0.013)	.001
	2011	0.049 (0.014)	.001
	2012	0.052 (0.017)	.003
	2013	0.037 (0.017)	.03
	2014	0.021 (0.017)	.22
	2015	0.084 (0.020)	<.001
	2016	0.080 (0.024)	.001
	Constant	5.042 (1.077)	<.001

^a^N/A: not applicable.

Estimates for all timepoints, from 3 years before to 3 years after a breach, were not significant. These estimates suggest that breaches were not associated with value added.

Total assets, total labor, IT capital, IT labor, and the number of beds were positively associated with value added. The number of individuals affected and the case mix index were not associated with value added. Breach type and breach location were associated with value added.

## Discussion

### Principal Findings

Hospitals’ breach responses increase the financial burden on hospitals. The efforts to repair the damages from a data breach increase direct and indirect costs and may divert resources from improving patient quality of care. Health care data breaches reported to HHS, which includes breached health plans, physicians, and business associates in addition with hospitals, have grown from 329 in 2016 to 642 in 2020 [19]. Hospital data breaches were reported to increase hospital advertising expenditures [20] and IT spending [21] to remedy the damage due to a data breach.

Breached hospitals were larger in size, reflected in higher value added, total assets, and total labor, which is consistent with previous findings [7,22]. Larger hospitals have more access points, devices, and staff that could be breached, both intentionally and erroneously. Thus, the risk of a data breach is proportional to an organization’s size.

However, data breaches were not associated with a reduction in productivity; that is, we did not observe a significant relationship between breaches and hospital productivity measured as the value added. Hospital productivity was resilient against the shocks from a data breach. We hypothesized that the financial cost and disruption associated with data breaches may decrease hospital production, but our results suggest that hospital productivity was unaffected. The stability in hospital productivity also implies that patient demand for hospital services was inelastic to data breaches. The remediation efforts and advertising to repair the reputation of the breached hospitals may have contributed to the steady demand.

Moreover, there are at least 2 more reasons to explain these results. First, there is incredible heterogeneity in the information type from a breach. For example, the release of patient records is likely to undermine the reputation of a hospital, whereas malware attacks are more likely to reduce cash flow rather than the hospital’s reputation. The effects of different attack types may take longer to manifest for hospitals. Second, while many breaches take place without knowledge, as reflected by the large uncertainty about hospital vulnerabilities, those that detect incidents may not have an incentive to report the full financial impact [23]. Most hospitals are not-for-profit organizations. We are not aware of a federal or state law that requires not-for-profit organizations to disclose data breaches in their financial statements. The Sarbanes–Oxley Act of 2002 requires publicly traded firms to disclose data breaches, but investor-owned hospitals account for a small fraction of all hospitals.

Emphasis should be laid on the security training of health care workers. Treating patients and saving lives are the highest priority for health care workers, which makes them cautious in handling hospitals’ security regulations and policies. However, nearly one-third of the health care workforce had never received cybersecurity-related training [24]. This lack of awareness results in improper handling and storage of patient files, with increasing usage of mobile devices. The most frequent breach type in our study sample was data theft, and the most frequent breach location was desktop and laptop computers. In health care, internal human error and misuse occur much more frequently than external attacks such as those that involve hacking [25]. Thus, to reduce the risk of a hospital data breach, health care workers should be trained in cybersecurity.

Hospitals are an attractive target for cyber attackers, and these attackers are affecting hospitals by using ransomware [26,27]. While our study data do did not capture ransomware attacks, these are considered much more disruptive than data breaches. To mitigate the threat, health care organizations should share threat information, experiences, and best practices to build the appropriate security architecture.

### Limitations

Our analysis included reported health data breaches, which affected more than 500 individuals from 2012 to 2016; however, this is not an exhaustive list of data breaches. Smaller data breaches that affect fewer than 500 individuals are not published by HHS; hence, such breaches were excluded from our study. There is a nontrivial number of unpublished small data breaches [28]; however, such breaches tend to be less costly for organizations to remediate. There are various types of data breaches, and given the heterogeneity in potential breach effects, our small sample of breached hospitals limited the precision of our model estimates.

### Conclusions

Hospital productivity was resilient against the shocks from a data breach between 2012 and 2016. The productivity trend of breached hospitals remained parallel with that of control hospitals in the years before the breach. Thereafter, the productivity of breached hospitals did not diverge significantly in the years after the breach. Nonetheless, data breaches continue to threaten hospitals today; therefore, health care workers should be trained in cybersecurity to mitigate these disruptions.

## Abbreviations

HHS: US Department of Health and Human Services
IT: information technology
OCR: Office for Civil Rights
OSHPD: California Office of Statewide Health Planning and Development
